# Splenocolic fistula in a patient with diffuse large B-cell lymphoma: A case report and review of literature

**DOI:** 10.3389/fsurg.2022.979463

**Published:** 2022-09-20

**Authors:** Tonia Luca, Mauro Sergi, Onella Coco, Emanuela Leone, Giulia Chisari, Paolo Fontana, Sergio Castorina

**Affiliations:** ^1^ Department of Medical, Surgical Sciences and Advanced Technologies G.F. Ingrassia, University of Catania, Catania, Italy; ^2^ Department of Experimental Oncology, Mediterranean institute of Oncology, Viagrande, Catania, Italy; ^3^ Fondazione Mediterranea G.B. Morgagni, Catania, Italy

**Keywords:** fistula, atypical presentation, splenomegaly, lymphoma diagnosis, diffuse large B-cell lymphoma

## Abstract

A fistula that connects the bowel to other organs, such as the urinary bladder or small intestine, is a relatively frequent complication, often associated with inflammatory diseases such as diverticulitis, Crohn's disease, colorectal cancer, or lymphoma. Splenocolic fistula is an extremely rare condition described in the literature. It can occur in cases of splenic tumors, including splenic diffuse large B cell lymphoma. We report the case of an 82-year-old man who presented with melaena, worsening asthenia, hypotension, and abdominal pain in the left flank and the ipsilateral lumbar region. Ultrasound and computed tomography documented splenomegaly, thickening of the splenic flexure of the colon, and the presence of a fistulous passage between the colon and the splenic hilum. The diagnosis of lymphoma was made following laparotomy and caudal splenopancreatectomy. Due to the aggressive clinical behavior of this type of lymphoma, splenectomy is the main treatment in patients with splenomegaly, abdominal pain, and tumor expansion.

## Introduction

A colonic fistula is an abnormal tunnel that connects the colon to the skin or an internal organ. When abnormal communication occurs between the colon and spleen, a splenocolic fistula develops, a rare condition described in the literature (1). It has been described in association with malignancy (2), Crohn's disease (3), and pancreatitis (4).

Diffuse large B-cell lymphomas (DLBCLs) are lymphoid neoplasms characterized by rapid, aggressive growth, making up about 40% of B-cell malignancies (5). They are the most frequently diagnosed form in Western countries. The average age of onset is around 65 years, with a preference for the male sex. These neoplastic forms are often associated with a 3q27 translocation affecting the *BCL6* gene, but translocations involving the *BCL2* or *MYC* genes can also occur (6). Patients with DLBCLs are stratified into prognostic groups based on the International Prognostic Index (IPI), calculated in relation to predictor factors such as age, lactate dehydrogenase (LDH), sites of involvement, Ann Arbor stage, and ECOG performance status (7).

DLBCLs are characterized by extensive lymph node involvement, systemic symptoms, and, in 40% of cases, an extranodal localization (more often affecting the gastrointestinal system). Clinically, they determine palpable splenomegaly, abdominal pain, and signs of tumor expansion in cases of hepatic and splenic involvement (8). In the last decade, a remarkable improvement in long-term disease control and overall survival has been observed, with more than 80% of patients in remission 5 years after the treatment. The standard treatment of advanced DLBCL is a combination of chemotherapy plus immunotherapy. Usually, complete remission is observed after first-line treatment (9). Most relapses occur during the first 2 years after finishing treatment, and often, they are associated with a poor prognosis.

Intra-abdominal fistula formation is frequent. It is often due to inflammatory gastrointestinal diseases or malignant tumors. It is known that fistulas occur mainly in various kinds of lymphoma rather than carcinomas (10). We report here a rare case of splenocolic fistula in a patient with diffuse large B-cell lymphoma, highlighting the nonspecific clinical picture that can lead to a diagnostic and therapeutic delay.

## Case report

An 82-year-old man presented with worsening asthenia, hypotension, abdominal pain in the left flank and ipsilateral lumbar region, and melaena. He had been reporting for about 1 year the onset of physical discomfort, anemia, abdominal pain in the left quadrants, and tendency to be constipated. He had already undergone an esophagogastroduodenoscopy and a pancolonoscopy, which had not shown any significant pathologies. On physical examination, he was afebrile and had a tender abdomen on deep palpation on both the left side and the ipsilateral lumbar region. Laboratory tests were significant for leukocytosis (17,700 white blood cells per cubic millimeter), anemia (hemoglobin 10.50 mg/dl), and thrombocytosis (901,000 platelets per cubic millimeter). The patient was given fluid resuscitation and broad-spectrum antibiotics. Ultrasonography of the abdomen showed splenomegaly (DT 15.32 cm), presence of a hypoechoic area with a pyramidal shape (base to the splenic hilum and apex to the subcapsular site), and intralesional air, indicating the possible presence of a fistula. A thickening of the splenic flexure of the colon with a fistulous tract that infiltrated the hilum of the spleen was evident.

**Figure 1:**
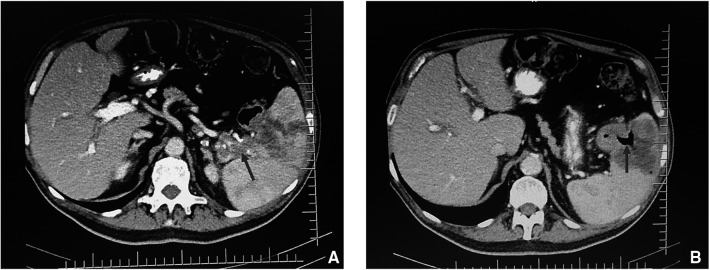
CT scan of the thorax and abdomen showing (**A**) suspicious fistulous tract between colon and spleen (arrow) and (**B**) air in the spleen (arrow).

**Figure 2:**
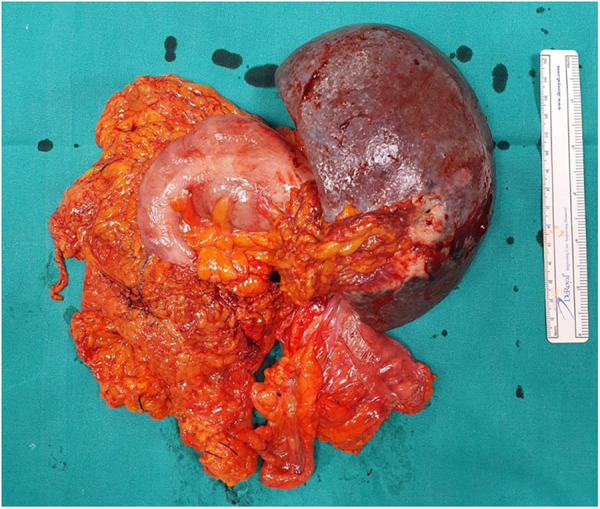
Surgical specimens containing splenic flexure, descending colon, and peripancreatic adipose tissue adhered and in close relationship with the medial surface of the spleen.

**Figure 3:**
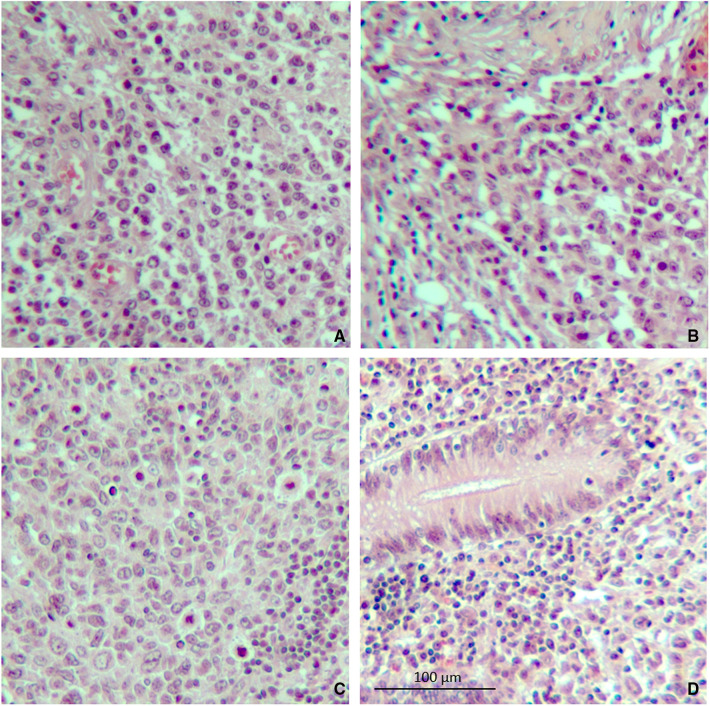
Microscopic specimen of the spleen and colon. (**A**) Atypical lymphoma cells were found in the spleen on hematoxylin–eosin stain. (**B**) Evidence of capsular invasion by lymphoma cells. (**C**) Splenic hilum lymph node containing lymphoma cells. (**D**) Lymphoma cells found in the colon wall near the splenocolic fistula (10× magnification).

An abdomen CT scan showed splenomegaly and concentric wall thickening of the descending colon near the splenic flexure, with fistulous communication between the colon and the medial surface of the spleen. A hypodense area with air bubbles was also evident (Figure 1). Because the mass infiltrated the splenic parenchyma at the hilum and the peripancreatic adipose tissue, the patient underwent splenectomy, distal pancreatectomy, and splenic flexure resection. A side-to-side anastomosis and an ileostomy were then fashioned in the right iliac fossa. The ileostomy was to protect the integrity of the side-to-side colo-colic anastomosis, which was performed under suboptimal conditions. Macroscopic examination showed a spleen measuring 18 × 11 × 8 cm. In the dome of the spleen, a 2 cm ulcerated whitish nodule was present. A solid whitish neoformation of 7 × 6 × 4 cm at the level of the splenic flexure was also evident (Figure 2). Histopathological examination of tissue samples confirmed that DLBCLextended to the splenic hilum, diffusely infiltrating the organ, the transverse mesocolon, the left flexure, and the adipose tissue of the pancreas tail (Figure 3).

**Figure 4:**
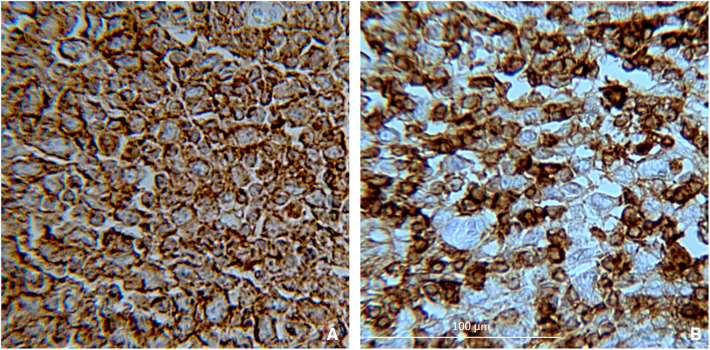
Positivity for (**A**) CD20 and (**B**) CD43, indicating that it is large diffuse B-cell lymphoma (20× magnification).

Immunohistochemical analysis revealed CD20, CD43 (Figure 4), CD22, PAX5, CD45, and VIM (data not shown) protein expression. The postoperative course was uneventful. A follow-up chest x-ray showed left-sided pleural effusion. The patient was submitted to pleural drainage, and the pleural fluid culture was positive for *Klebsiella pneumoniae*. Specific antibiotics were then administered. Once the infection had cleared, the patient left the clinical center and began chemotherapy in association with immunotherapy for cancer treatment, according to the Clinical Practice Guidelines. The regimen is called R-CHOP (Rituximab, Cyclophosphamide, Doxorubicin, Vincristine, and Prednisone, administered every 21 days for 6–8 cycles). The patient is still being treated. Three months later, an ileostomy closure surgery was done. The patient underwent laboratory tests and radiological examinations every 3/6 month for 1 year and is still undergoing cancer therapy.

## Discussion

DLBCL is classified within the non-Hodgkin lymphomas (NHLs), accounting for about 40% of all NHL cases worldwide (11, 12). It is also the most often diagnosed form in Western countries. The disease occurs more frequently in whites, young or old adults, with a male preponderance. It usually starts as a mass that grows quickly in a lymph node deep inside the body, such as in the chest or abdomen, or in a lymph node near the body's surface, such as in the neck or armpit. It can also start at a site other than a lymph node, such as bones, brain or spinal cord, and the intestines. It is characterized by the absence of generalized lymphadenopathy and bone marrow involvement. The diagnosis is based on the combined results of biopsy, processing, and microscopic examination to evaluate the morphology. Immunophenotyping, along with staining for B-cell markers, is usually performed (13). Clinically, DLBCL is characterized by rapid symptomatology with splenomegaly, dyspepsia, pain, and sometimes symptoms of intestinal obstruction. In cases of extranodal involvement, symptoms and signs can vary, depending on which organs or tissues are affected. They reflect the presence of a rapidly expanding tumor or infiltrate that produces symptoms specific to the organ of involvement, such as increased size, pain, and/or dysfunction. Treatment can be a combination of radiation and chemotherapy. Surgery is performed when there is damage to the liver and spleen.

The definition of primary extranodal lymphoma is controversial, especially in those patients who have both nodal and extranodal involvement. According to Krol et al. (14), primary NHL is considered extranodal when it originates at an extranodal site, even in the presence of disseminated disease, as long as the extranodal component is clinically dominant.

NHL can start anywhere in the body where lymph tissue is found, and for this reason, the digestive tract, where many organs have lymph tissue, is often involved. This is particularly true for DLBCL.

Intra-abdominal fistula formation is a rare event. Fistulas are usually the result of intra-abdominal inflammation or infection or the consequence of malignant disease, or they develop as a complication of abdominal surgery or trauma. Our search of the literature led to the finding of a previous report of colonic fistula formation, a gastrocolic fistula (15), and, subsequently, two more recent cases of fistula connecting the colon and spleen (16, 17). Many studies documented the tendency of the colon to fistulize to contiguous organs, such as the bladder (colovesical fistula), vagina (colovaginal fistula), and intestinal loops (coloenteric fistula). Other organs, such as the uterus, gallbladder, and spleen, are rarely involved (18–21).

Fistula formation can be a consequence of different pathologies, such as Crohn's disease (22), diverticular disease (23), colorectal cancer (4, 24), and lymphoma (25).

It is known that a fistulization is a rare event in malignancies such as gastric or colonic cancer (26). The development of fistulas in association with gastrointestinal lymphoma, although rare, is more common than in carcinoma because of the absence of a desmoplastic reaction (27). Splenic involvement rarely occurs. Treatment usually consists of the resection of the segment of the colon and the other affected organs. Unlike colovesical and colovaginal fistulas, splenocolic fistula is a rare clinical entity, with few cases reported in the literature. As for the most common forms, it can be secondary to various inflammatory and neoplastic conditions, including complicated diverticular disease, locally advanced colorectal cancer, lymphoma (10), or pancreatitis (28). Fistulas between the colon and spleen, even if rare, are often associated with severe Crohn's disease (3, 29).

Both in splenocolic fistula and other cases of colic fistulas associated with malignancies, necrosis is often thought to be the ultimate cause of fistula formation. Necrosis is accompanied by ulceration and extensive coagulation. In most cases, surgery, after preoperative imaging, allows removal of the fistulous tract by resecting the organs, or the affected portions of them, involved in the pathological process.

In our case, the patient presented with a rare splenocolic fistula. Surgical intervention was urgent and particularly useful to diagnose the fistula associated with DLBCL and to confirm the splenic localization. Similar cases have been reported in the literature. In particular, Katayama et al. described a case of a fistula of the colon in non-Hodgkin's lymphoma of the spleen (30). Natschitz et al. described a colosplenic fistula that complicated immunoblastic lymphoma.

In conclusion, we report an exceedingly rare condition in which the striking feature was the presence of a splenocolic fistula associated with DLBCL in an elderly patient. Our findings suggest that lymphoma cells had infiltrated from the spleen to the colon. As with any severe chronic pathology, early detection and recognition of the disease process were necessary to prevent complications. It is crucial to consider splenocolic in the differential diagnosis when a patient present with sepsis, splenomegaly, abdominal pain, and tumor expansion.

## Data Availability

The original contributions presented in the study are included in the article/Supplementary Material, further inquiries can be directed to the corresponding author/s.
